# Regression and Disappearance of Accelerated Nodulosis with Adalimumab

**DOI:** 10.31138/mjr.031125.ara

**Published:** 2026-06-01

**Authors:** Georgios A. Drosos, Anastasia K. Zikou, Paraskevi V. Voulgari, Alexandros A. Drosos

**Affiliations:** 1 Department of Rheumatology, School of Health Sciences, Faculty of Medicine, University of Ioannina, Ioannina, Greece;; 2 Department of Radiology, Medical School, University of Ioannina, Ioannina, Greece

**Keywords:** RA, rheumatoid nodules, accelerated nodulosis, methotrexate, adalimumab

Accelerated nodulosis is the rapid progression of pre-existing rheumatoid nodules (RN), occurring in patients with rheumatoid arthritis (RA), treated with methotrexate (MTX). There is still controversy regarding the optimal management of patients suffering from accelerated nodulosis.^1,2^ A 71-year-old woman with a 20-year-history of seropositive RA presented with pain, swelling and stiffness of both hands. Past medical history was positive for hypertension treated with losartan 20mg/day. She was single and no smoker. The patient did not receive any treatment except for analgesics and occasionally steroids until 10 years ago, when she was treated with MTX 15mg/weekly and prednisone 5mg/day. After six months of therapy, she felt well but she noticed the presence of RN, two on the first digit and one on the third digit of the right hand. She continued the treatment with MTX and the last year she noticed that the RN were progressed and increased in number and became painful. Clinical examination revealed swelling and tenderness over the metacarpophalangeal joints (MCPs) bilaterally, with multiple RN affecting almost all digits in the volar (**Figure 1A**) and palmar (**Figure1B**) areas, along with ulnar deviation of both hands (**Figure 1A–B**). Laboratory tests showed C-reactive protein (C-RP) of 30mg/l (normal values <6), erythrocyte sedimentation rate (ESR) 58mmHg/h, rheumatoid factor 205U (normal value <10), while anti-citrullinate protein antibodies (ACPA) and anti-nuclear antibodies (ANA) were negative. The rest of laboratory tests were within normal limits. Hand and wrist x-rays showed severe destructive changes affecting both wrists, all MCPs and proximal interphalangeal (PIPs) joints bilaterally and severe subluxations (**Figure 2**). The dose of MTX decreased to 10mg/w and adalimumab (ADA) 40mg subcutaneously, every two weeks was added. After two months she had a substantial clinical and laboratory response. The acute phase reactants were normalized, while the number and size of RN were unaffected. However, one year later all RN were regressed and disappeared with significant clinical improvement (**Figure 3A–B**).

**Figure 1.**
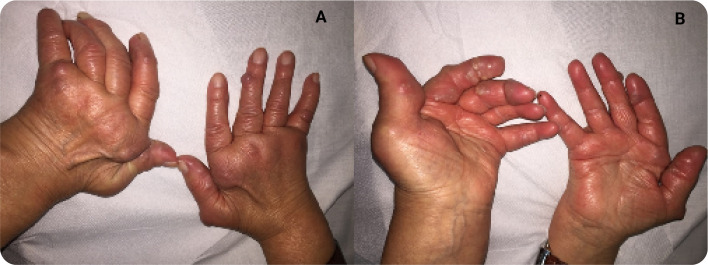
Swelling of all metacarpophalangeal joints of the hands, muscle atrophy and ulnar deviation are evident bilaterally. Note also the multiple subcutaneous rheumatoid nodules in various sizes affecting almost all digits in volar (**A**) and palmar (**B**) areas.

**Figure 2.**
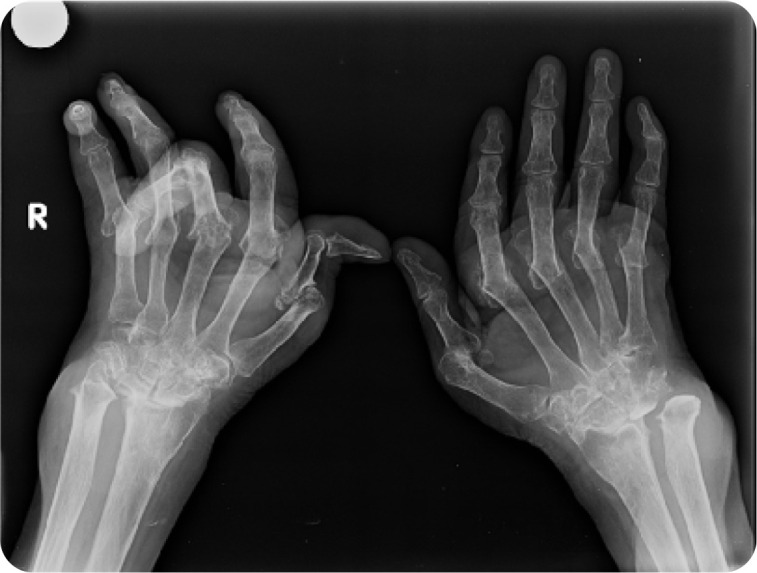
**(above).** Hands and wrists x-ray of the same patient. Note the severe destructive changes affecting all carpal bones, all metacarpophalangeal and proximal interphalangeal joints bilaterally. Severe subluxations of metacarpophalangeal, proximal interphalangeal and distal interphalangeal joints, along with cysts formations are evident.

**Figure 3.**
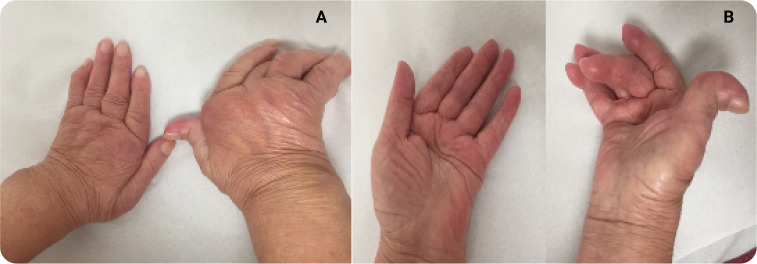
**(below).** The same patient as in Figure 1, after one year treatment with adalimumab. Note the regression and disappearance of all rheumatoid nodules in both hands in volar (**A**) and palmar (**B**) areas.

MTX is the fundamental agent among conventional synthetic disease-modifying Antirheumatic Drugs (csDMARDs), for RA treatment. However, MTX may induce accelerated nodulosis (MIAN), which occurs rarely in patients treated with this agent.^3^ The development of MIAN occurs mostly in RA patients, but also in psoriatic arthritis, juvenile idiopathic arthritis and lupus patients.^2–4^ Accelerated nodulosis has been observed during treatment with other csDMARDs, such as leflunomide. It has been also reported with the use of other immunosuppressive drugs such as, azathioprine as well as biologic (b) DMARDs like tocilizumab and infliximab.^2^ The mechanism of MIAN is not well known. It seems that MTX induces adenosine production from inflammatory monocytes, which stimulates adenosine A1 receptors enhancing giant cells formation, in vitro.^5^ As regards the histological findings of MIAN, they are similar to RA related RN, being composed of three zones: a) inner central necrobiotic zone, b) surrounded zone with palisading cells and c) outer zone consisting with perivascular infiltration of chronic inflammatory cells.^6,7^ The cumulative dose of MTX for the development of accelerated nodulosis ranges from 90 to 7200mg. Risk factors for accelerated nodulosis include smoking, high titres of RF and /or ACPA and the human leucocyte antigen (HLA) DRB1 0401 haplotype.^2,6^ There are no guidelines for MIAN management. The primary treatment is the tapering or discontinuation of the offended agent. The administration of steroids, hydroxychloroquine, colchicine, sulfasalazine, D-penicillamine has been reported to reduce the size and the number of nodules.^2,3 6^ Our patient has an established, seropositive, untreated RA for many years. She received MTX and she noticed deterioration of the RN during treatment. In our patient MIAN was completely regressed, and all RN disappeared after ADA therapy. In conclusion, physicians must be aware of, recognize and treat appropriately this adverse event during MTX therapy. To our knowledge, this is the first report of disappearance of MIAN after ADA therapy.

## DECLARATION

This study was performed in accordance with the Helsinki Declaration of 1964 and its later amendments. All presented material is published after written consent of the patients, although sensitive data and personal details are not included in the publication.
